# Characterization of LpGPAT Gene in *Lilium pensylvanicum* and Response to Cold Stress

**DOI:** 10.1155/2015/792819

**Published:** 2015-02-01

**Authors:** Shao-kun Sun, Ni-na Yang, Li-jing Chen, Muhammad Irfan, Xing-hua Zhao, Tian-lai Li

**Affiliations:** ^1^Key Laboratory of Agricultural Biotechnology of Liaoning Province, College of Biological Science and Technology, Shenyang Agriculture University, 120 Dongling Road, Shenyang, Liaoning 110866, China; ^2^Key Laboratory of Protected Horticulture, College of Horticulture, Ministry of Education, Shenyang Agriculture University, 120 Dongling Road, Shenyang, Liaoning 110866, China; ^3^Liaoning Academy of Agricultural Sciences, Shenyang 110161, China

## Abstract

LpGPAT was obtained from *L. pensylvanicum* using RT-PCR and rapid amplification of cDNA ends. The cloned full-length cDNA was 1544 bp; it encoded 410 amino acids and had a molecular size of 46 KDa. The nucleic acid sequence analysis showed that it shared high homology with other known GPATs. SMAT result suggests that there is a PlsC that exists in 176-322 amino acid sequence of LpGAPT; it means LpGPAT protein is a member of the family of acyltransferase and has acyltransferase enzymatic activity. Result of real-time quantitative PCR and semiquantitative PCR support LpGPAT gene is definitely induced by low temperature stress.

## 1. Introduction

*Lilium pensylvanicum* belongs to Liliaceae,* Lilium*, which originates in North China including Hebei, Heilongjiang, Jilin and Liaoning provinces, North Korea, Japan, Mongolia, and Russia [1]. It is one of the best parents to cultivate novel anti-cold* Lilium* varieties for strong cold-resistance. Besides, it possesses significant resistance against heat, drought, alkali, salt, sand burial, and aeolian erosion. It even can survive under contions with temperature ranging from −70°C to −40°C and annual rainfall less than 200 mm [2]. Hence, it is precious and valuable rescorce for drought-resistance and cold-resistance, as well as ideal material for resistance studying.

Hypothermia is the main factor limiting plant growth, development, and geographical distribution. One adaptation of plants to temperature stress is by biomembranes, especially the effect of plasma and thylakoid membranes. Plants can regulate the level of unsaturated fatty acids to cold through the acyl lipid desaturase and glycerol-3-phosphate acyltransferase (GPAT) activity [3]. A large number of studies indicate that the saturation degree of phosphatidyl glycerol (PG) in membrane is closely related to cold tolerance in plants. While GPAT is the first acyltransferase during the PG synthesis and it initiates the reaction by the acylation of the glycerol-3-phosphate. The substrate selection of GPAT plays a key role to determine the unsaturation degree of the PG molecules [4]. Different GPATs in plants with low-temperature tolerance have different substrate selectivity of acyl, that is, GPATs from plants with strong cold-resistance prefer oleic acid, while GPATs from cold-sensitive plants utilize the palmitic acid and oleic acid equally [5]. Such selective difference of GPAT to substrate affects the saturation level of the PG in plant membrane, which consequently determines the plant resistance to low temperature [6]. Later, the study in the relationship between plant cold-tolerance and the fatty acid saturation in membrane achieves a breakthrough [4, 7]. The fatty acids (saturated and unsaturated) of PG in the chloroplast membrane have influence on the synthesis of cold-sensitive plants [8].

Hypothermia is inevitably a limitation to achieve annual production for the climate feature of China and behind hand horticulture facilities with contrast to Europe and America. Therefore, it is necessary to study the cold tolerance of* L. pensylvanicum *in order to save energy, reduce the costs of winter cultivation and storage, and improve the stress-resistance to achieve the large-scale cultivation of* L. pensylvanicum*. Additionally, the bad natural environment and weather of wild* L. pensylvanicum* confers its very strong cold tolerance. Hence, there are abundant excellent cold-resistant-related genes. Exploration and utilization of these genes have important significance for the research on cold tolerance of plants and improvement. In this study, the conservative fragment of* L. pensylvanicum* GPAT gene was obtained by RT-PCR. The whole cDNA of the LpGPAT gene was cloned by RACE, which was then analyzed for expression. This study will supply some basis for the deep research on the cold-resistance of* L. pensylvanicum*.

## 2. Materials and Method

### 2.1. Plant Materials and Pretreatment

*Lilium pensylvanicum* bulbs were kindly provided by Daxinganling Forestry Bureau, Inner Mongolia province, China, which had been domesticated from wild species over three years.* L. pensylvanicum* possessed strong cold tolerance that was stored with 10 cm covered soil in winter and could survive with 50°C to below 0°C.

The bulbs from* L. pensylvanicum* were separated and washed by water, then soaked in 75% ethanol for 30 s, and then put in 0.1% HgCl_2_ for 10 min. Subsequently, they were rinsed with sterilized water 3 times. At last, they were placed on dry sterile filter-paper for use. The sterile bulbs were cultured on MS medium adding agar powder (7 g L^−1^) and sucrose (30 g L^−1^), pH 5.8. The fluorescent light was on 14 h everyday with light intensity of 1000~1200 lx. The temperature was from 23 to 26°C. Two months later, the adventitious buds were removed from the bulbs and placed in 4°C for 2 h, 4 h, 6 h, 8 h, 12 h, 24 h, 48 h, and 72 h, which were later used for RNA extraction or were immediately frozen in liquid N_2_ and stored in ultra-low temperature freezer at −80°C until needed.

Total RNA samples used for cDNA first strand synthesis were extracted from the above treated fresh young leaves. After that semiquantitative PCR and real-time quantitative PCR reactions were performed using these samples to detect relative differential expressions of LpGPAT gene under different low temperatures stress.

The RNA extraction kit, vector PGM-T, miniplasmid-DNA extraction kit, DNA polymerase, dNTPs, DNA Marker D2000, and* E. coli Top 10* used in the experiment were purchased from Tiangen Biotech Co., Ltd. (Beijing, China). The agarose gel and DNA Gel extraction kit were bought from AxyGen (Beijing, China). M-MLV Reverse Transcriptase was from Promega (Beijing, China). All the other chemicals were of analytical grade. All the primers were synthesized by SBS Genetech Co., Ltd. (Beijing, China).

### 2.2. RNA Extraction and First-Strand cDNA Synthesis

Leaves were ground in liquid N_2_ with a mortar and pestle. Total RNA was extracted from samples with the plant tissue RNA extraction kit following the manufacturer's instructions. The RNA quality was assessed by using 2 *μ*g of the total RNA on a 1.2% agarose gel. The total RNA was stored at −80°C. cDNA synthesis reactions were performed with M-MLV reverse transcriptase according to the manufacturer's instructions.

### 2.3. Isolation of the Middle Fragments of the LpGPAT Genes

The homologous primers for middle segments were designed based on conserved sequences of GPAT genes from some plants in NCBI GenBank (GPATF1, F2, R1, and R2, Table 1). PCR reactions were performed in a total reaction volume of 25 *μ*L containing 50 ng of genomic DNA, 1× Taq DNA polymerase buffer, 1.5 mM MgCl_2_, 0.5 *μ*M each primer, 200 *μ*M each dNTP, and 1U Taq DNA polymerase. The program for PCR amplification was as follows: initial denaturation at 94°C for 5 min, 35 cycles of 94°C for 30 s, 53°C for 45 s, 72°C for 60 s, and a final extension at 72°C for 10 min. The PCR products were separated on 1.0% agarose gels, and then the targeted DNA fragments were recovered and cloned into the pGEMT-Easy vector. The ligated products were transformed into* Escherichia coli* (DH5*α*) cells and the resulting plasmids were used as a sequencing template.

### 2.4. Amplification of the Complete Coding Sequences of the LpGPAT Genes

Primers for 5′- and 3′-end cDNA amplification were designed based on the middle fragment sequences (Table 1). The 3′ and 5′ sequences of cDNA were obtained by RACE with the 3′- and 5′-RACE System for Rapid Amplification of cDNA Ends (Invitrogen, USA). The PCR products were cloned to pMD18-T vector and sequenced. Based on the nucleotide sequences of the 5′- and 3′-RACE products, primers LpGPAT 1F, LpGPAT 1R, LpGPAT 2F and LpGPAT 2R (Table 1) were used for the amplification of the complete coding sequences of* GPAT* genes.

### 2.5. Semiquantitative RT-PCR Analysis

Semiquantitative RT-PCR was performed using gene-specific primers for EGPATF and EGPATR. As an internal control, a fragment from white ash actin gene was amplified using the actinF and actinR primers (Table 1). The PCR amplification was programmed as initial denaturation at 94°C for 4 min, 35 cycles of 94°C for 30 s, 56°C for 1 min, 72°C for 1 min, and a final extension at 72°C for 10 min. Amplified fragments were detected by electrophoresis using 1.5% (w/v) agarose gels.

### 2.6. Bacterial Expression of LpGPAT

According to the full-length cDNA sequence analysis of the cloned LpGPAT gene and the prokaryotic expression vector pET30a (+), we designed a pair of primers which were in accordance to the cDNA ORF in LpGPAT as follows:

 YGpat1 F (5′-GGTACCATGCTACGTCGGGAACCG-3′),
 YGpat1 R (5′-GAATTCTCATGGTTCTGAGAGAGAGATAG-3′).

*Eco*RI and* Kpn*I restriction sites were added in the primers (underlined part). A recombinant plasmid pMD18T-LpGPAT and the expression vector were digested with* EcoR*I and* Kpn*I. Then target gene was ligated to pET30a (+), which was later used to transform* E. coli* BL21 competent cells. The correct colony was identified by screening medium with Kan and PCR. Finally, the recombinant* E. coli *was cultured in liquid medium and 1 mol/L IPTG was added to induce the protein expression with different time.

### 2.7. Bioinformatic and Phylogenetic Analysis

Primer Premier 5 software (http://www.Premierbiosoft.com) was used for all the primer designs. Sequences were aligned using DNAman 5.2.2 (http://www.lynnon.com) and SMART (http://smart.embl-heidelberg.de/) and CLUSTAL W 1.81 software [9]. The phylogenetic tree was generated based on the NJ (neighbour-joining) sequences distance method [10] and depicted and edited by MEGA 3.1 program [11].

### 2.8. Quantitative Real-Time PCR

Total RNA was extracted from the frozen leaves and bulbs in liquid nitrogen, removed from seedlings which treated with 4°C for 2 h, 4 h, 6 h, 8 h, 12 h, 24 h, 48 h, and 72 h, using an RNA extraction kit (Bioteke, China) according to the manufacturer's instructions. Reverse transcription was performed using MMLV reverse transcriptase (TaKaRa, China) with the Oligo-dT primer (TaKaRa, China). Quantitative real-time PCR was performed for cDNA amplification using SYBR Premix ExTaq (Takara Bio, Inc., Shiga, Japan) and primers listed in Table 1, on a Bio-Rad C1000 real-time system (Bio-Rad, Hercules, CA, USA), according to the manufacturer's instructions and applied international standards. For each PCR, 2 *μ*L cDNA obtained from 1 *μ*g RNA template was used. The thermal cycling conditions consisted of an initial denaturation step at 95°C for 30 sec and 40 cycles of the following 3 steps: denaturation at 95°C for 5 sec, annealing at 57°C for 30 sec, and elongation at 72°C for 30 sec. Actin was used as the internal control.

## 3. Results

### 3.1. Sequence Analysis of LpGPAT Genes

**Figure 1:**
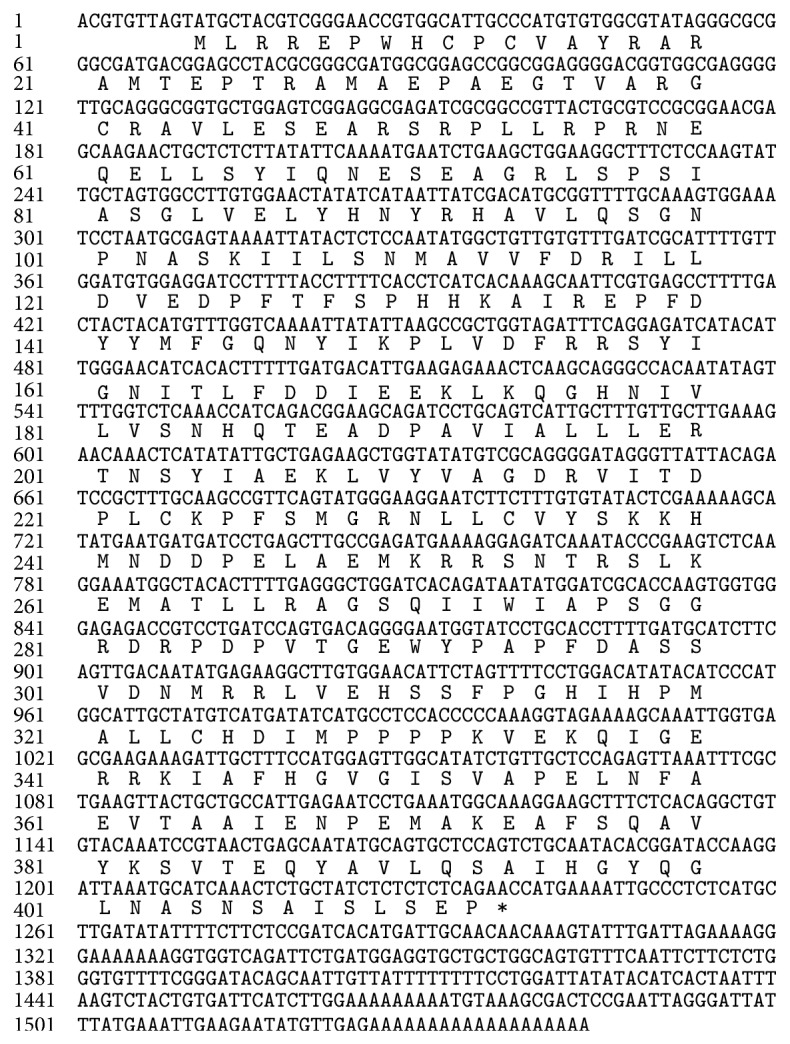
Nucleotide and deduced amino acid sequences of* LpGPAT.*

Initially, a fragment about 680 bp was amplified by RT-PCR. By analysis and comparison of the sequence, the fragment had the similar structures with those of known GPAT genes. This was followed by 3′- and 5′-RACE analysis, and two fragments, 750 and 500 bp, in sizes were obtained. Finally, the full-length cDNA of a LpGPAT gene was obtained by sequence assembly and reamplification. Sequence analysis revealed that the cDNA fragment was 1,544 bp in length, including an open reading frame (ORF) of 1,233 bp along with 311 bp 5′- and 10 bp 3′-untranslated sequences (Figure 1). This cDNA fragment that could encode 410 amino acids designated as LpGPAT had been submitted in GenBank (accession number JX524741).

### 3.2. Comparison of Amino Acid Sequences

**Figure 2:**
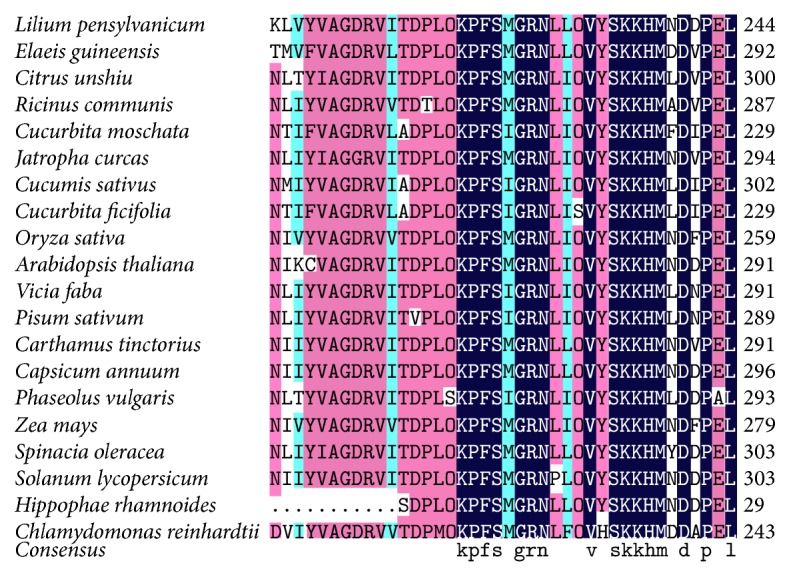
Comparison of the deduced amino acid sequences of* LpGPAT* with other known GPAT genes from 20 species. Deep (black) shading represents the identity of amino acid residues that was 100%; light (gray) shading represents the identity that was more than 75%.

The comparison of amino acid sequences showed thatLpGPAT had the similar typical primary structures to those of known GPAT genes from other plants such as* Arabidopsis thaliana*,* Oryza sativa*,* Zea mays*,* Elaeis guineensis*,* Solanum lycopersicum,* and* Chlamydomonas reinhardtii. *There were high levels of homology in amino acid sequences of all GPAT proteins from others (Figure 2).

**Figure 3:**
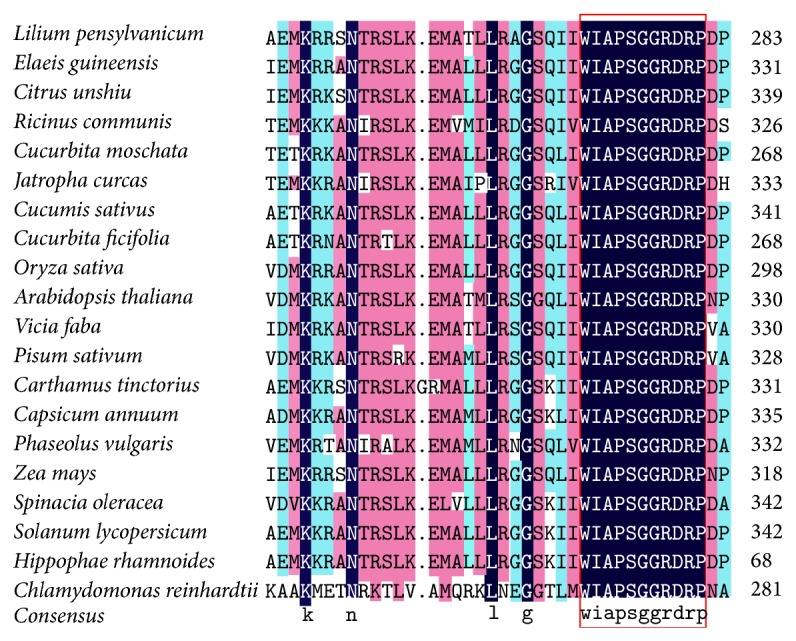
Alignment of deduced amino acid sequences from 20 plants GPAT cDNA. The domain of acyl-CoAs desaturase and the domain of acyl-ACPs family are in black box, respectively.

The coding regions begin from the amino acids MALK in all genes from woody plants. The conserved regions of all genes begin from about 40th amino acid residues (WIAPSGGRDRP) (Figure 3). All GPAT amino acid sequences have two conserved domains belonging to the acyl-CoAs desaturase family and the acyl-ACPs family, respectively. But they were also different from each other by some substitutions, insertions, and/or deletions involving single amino acid residues or motifs. The main regions of variability were regions about front 40 amino acid residues of N-terminal domains.

### 3.3. Semiquantitative RT-PCR Analysis

**Figure 4:**
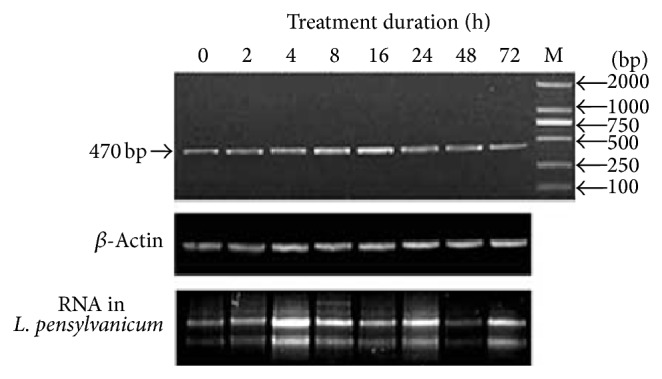
Expression of* LpGPAT* in* L. pensylvanicum* under different cold induced times at 4°C.

Semiquantitative RT-PCR was employed to confirm the expression patterns of the LpGPAT gene in different induction times. The result in this study showed that low temperature could induce the expression of LpGPAT. The expression analysis ofLpGPAT gene showed that low temperature could induce the expression of the gene in a short time. With the prolonged cold induction, the expression increased firstly and then decreased, a lot of expressions were found after 4 h, peaked at 16 h, and then decline gradually and at 72 h basically the same level in keeping with 0 h. (Figure 4).

### 3.4. Prokaryotic Expression Analysis of LpGPAT Gene

**Figure 5:**
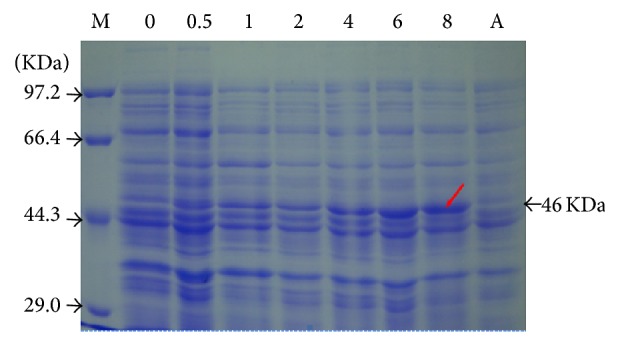
The expression of* LpGPAT* protein in* E. coli *BL21 by SDS-PAGE analysis.

The recombinant prokaryotic expression vector pET-LpGPAT was checked by enzyme digestion using* Eco*R I and* Kpn* I, which was later transformed into* E. coli* BL21. The correct transformants were cultured and expressed the LpGPAT proteins by adding IPTG. A predicted extra protein band of 46 kDa was observed in the transformants, while no such band was seen in the negative controls (Figure 5). Besides, the expression level increased with the treatment time from 0 to 8 hours.

### 3.5. Evolutionary Relationship Analysis

**Figure 6:**
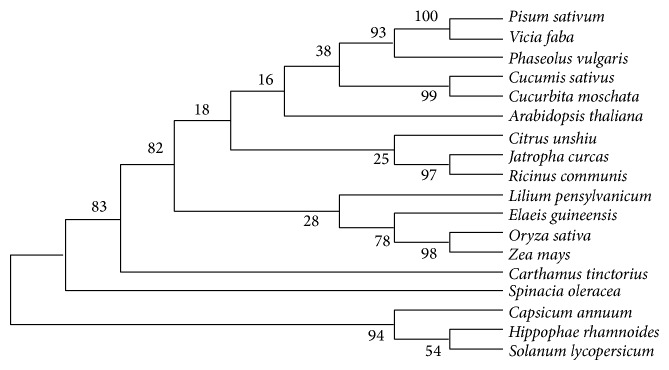
Phylogenetic analysis of amino acid sequences of LpGPAT from* Lilium* and other plants in GenBank database.

Evolutionary relationship was determined by sequences of GPATs obtained from BLAST and alignment using Clustalx 1.83 software. A tree was constructed by Mega 4.0 software using neighbour-joining method (Figure 6). We found that* L. pensylvanicum* was in the cluster with* E. guineensis*,* O. sativa, *and* Z. mays,* and then in the cluster with* Solanum lycopersicum*,* Capsicum annuum*,* C. tinctorius*,* C. moschata*,* Cucumis sativus*,* Arabidopsis thaliana,* and* C. reticulata*, and finally with* Spinacia oleracea* and* Chlamydomonas reinhardtii*. In conclusion, the result of the cluster analysis showed that the cloned LpGPAT gene in this research was genetically closer with the monocots, but farther with the dicots and farthest with the algae. Such a classification was also consistent with traditional plant taxonomy.

### 3.6. Protein Function Domains of LpGPAT

**Figure 7:**
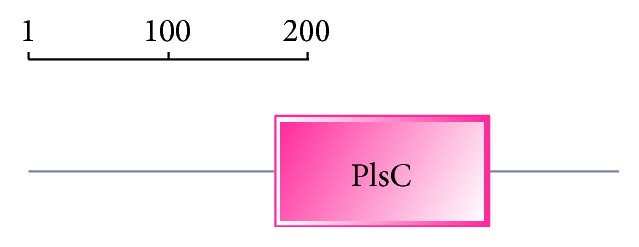
Prediction of protein domains in LpGPAT protein.

Protein domains of LpGPAT was predicted by online tools SMART (http://smart.embl-heidelberg.de/). The result suggests that there is a PlsC exist in 176–322 amino acid sequence of LpGAPT (Figure 7). PlsC is special structure domain of the acyltransferase, that can promote the synthesis of phospholipids and acyl transfer. It means LpGPAT protein is a member of the family of acyltransferase, has acyltransferase enzymatic activity.

### 3.7. LpGPAT Expression Pattern Analyses

**Figure 8:**
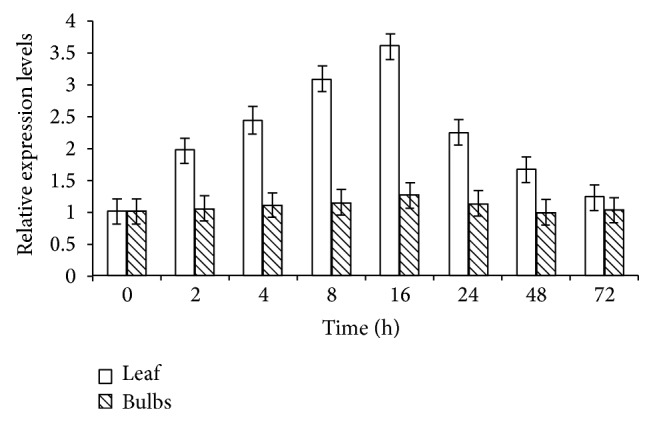
Relative expression levels of LpGPAT in leaf and bulbs from* L. pensylvanicum* under low temperature.

Real-time quantitative PCR reactions were performed using fresh young leaves and bulbs from tube seedlings to detect relative differential expressions of LpGPAT gene under low temperatures stress, in order to understand the expression pattern of LpGPAT gene from* Lilium pensylvanicum* leaves and bulbs, so as to further reveal its function and cold-resistant mechanism. The results show that LpGPAT expression levels in the leaf were significantly accumulated within 2 h after initiation of cold treatment and continued over the next 14 h and reached the maximum at 16 h. then the expression level decreases obviously. After 72 h chilling stress, LpGPAT expression levels have been falling, is slightly higher than that in control. However, after treatment, the changes of transcript levels in the bulbs were not obvious as that in leaf, and only faintly expressed in the bulbs (Figure 8).

The changes of LpGPAT expression level using real-time quantitative PCR are generally consistent with that of semiquantitative PCR, which both show that expression of LpGPAT is dramatically modified with different ranges of chilling stress. These results support that LpGPAT gene is definitely induced by low temperature stress.

## 4. Discussion

The low temperature injury is often associated with changes in the membrane lipid bilayer of one or more cellular membrane systems. Several mechanisms have been put forward as possible adaptive changes in membrane lipids in response to low temperature [12]. 3-Phosphate glycerol-acyltransferase is the key enzyme in biosynthesis of unsaturated fatty acids in the lipid. GPAT catalyses the first step of triacylglycerol synthesis and may be limiting for PA production. The fatty acid composition of the sn-1 position of cytoplasmic glycerolipids is also determined by the substrate specificity of GPAT [6]. Previous research has indicated the existence of a relationship between the expression of GPAT and chilling tolerance in many model plants [13, 14]. However, the function of the glycerol-3-phosphate acyltransferase gene in* Lilium* in response to chilling stress is still unclear. In this research, a LpGPAT gene was cloned from* L. pensylvanicum *using RT-PCR, 3′RACE, and 5′RACE techniques for the first time. The LpGPAT gene fragment in this study encodes 420 amino acids having molecular weight of 46 kDa which was higher than earlier reports. Gupta et al. [15] reported LlaGPAT gene fragment which encodes 370 amino acids with molecular mass of 41.2 kDa. Further homology and BLAST analysis of LpGPAT with other GPAT genes from* C. reticulata*,* Lagenaria siceraria*,* R. communis*,* C. moschata*,* Zea mays *L., and* C. tinctorius* confirmed the result. In addition, the amino acid sequencing revealed that the LpGPAT was homologous with those from cold-sensitive and cold-tolerant plants, suggesting that LpGPAT was related with cold resistance in plants. The results of this study revealed that low temperature could induce the expression of LpGPAT. Sui et al. [16] achieved higher levels of expression by exposing tomato leaves under 4°C for 4 h. The expression level got to the top after induction time of 3 h and it began to decline after 16 h, indicating that plants started fast LpGPAT expression under low temperature stress. Under the optimized conditions for the cultivation of recombinant* E. coli*, only the IPTG induction time was optimized during the experiment. Although the recombinant LpGPAT protein expression increased along with the induction time, the expression efficiency of the soluble protein was not very high overall. The probable reason for that might be caused by the induction temperature of 37°C. Perhaps some proteins accumulate to form inclusion bodies, and the concentration of IPTG (1 mM) might induce protein expression too fast which could result in formation of inclusion bodies of the expressed protein but no effective folding into active form. In the future, we will optimize the dose of inducing agent and the culture temperature to get a large scale of soluble protein for functional studies, as well as high expression of active purified protein.

## 5. Conclusion 

In this research, LpGPAT was obtained from* L. pensylvanicum*. The cloned full-length cDNA was 1544 bp with 5′-UTR of 10 bp, an open reading frame of 1233 bp, 3′-UTR of 311 bp, and poly(A) tail of 19 bp. The cDNA encoded 410 amino acids and had a molecular size of 46 KDa. The nucleic acid sequence analysis showed that it shared high homology with other known GPATs.

## Figures and Tables

**Table 1:** The sequences and use of the primers.

Primer name	Sequences (5′ → 3′)	Primer use
GPATF1	ATATTGC(T/A)G(A/C)AGGAATGGA(G/A)GA	For middle fragments
GPATF2	AATGGA(G/A)GA(A/G)(C/A)TGTAT(C/T)AGAA(T/C)TA	For middle fragments
GPATR1	GGAGG(G/A)GGCAT(A/G)AT(A/G)TCAT	For middle fragments
GPATR2	G(A/C)CCAGG(G/A)ACACCAG(C/A)ATGTT	For middle fragments
LpGPAT F1	TAAGCCGCTGGTAGATTTCA	For 3′RACE of LpGPAT
LpGPAT F2	TCTCAAACCATCAGACGGAAGC	For 3′RACE of LpGPAT
LpGPAT R1	AAGTGTGATGTTCCCAATGTATGA	For 5′RACE of LpGPAT
LpiGPAT R2	GTGGCCCTGCTTGAGTTTCT	For 5′RACE of LpGPAT
LpGPAT R3	TCTGCTTCCGTCTGATGGTTTGA	For 5′RACE of LpiGPAT
LpGPAT R4	CAAAGAAGATTCCTTCCCATACTG	For 5′RACE of LpGPAT
EGPATF	GCAGGGGATAGGGTTATTACAGA	For expression of LpGPAT
EGPATF	AGGATTCTCAATGGCAGCAGTA	For expression of LpGPAT
actinF	TCCTCTTCCAGCCTTCTTT	For control gene
actinR	TTCCTTGCTCATACGGTCA	For control gene
ActinF	GTCCATCCATCGTCCACAG	For real-time quantitative PCR
ActinR	CCTCAACAAGCCACCTACC	For real-time quantitative PCR
GPN1	CTCCACCCCCAAAGGTAGAAA	For real-time quantitative PCR
GPN2	TGGCAGCAGTAACTTCAGCGA	For real-time quantitative PCR
